# Interleukin 17 enhances bone morphogenetic protein-2-induced ectopic bone formation

**DOI:** 10.1038/s41598-018-25564-9

**Published:** 2018-05-08

**Authors:** M. Croes, M. C. Kruyt, W. M. Groen, K. M. A. van Dorenmalen, W. J. A. Dhert, F. C. Öner, J. Alblas

**Affiliations:** 1 Department of Orthopaedics, University Medical Centre Utrecht, Heidelberglaan 100, 3584 CX Utrecht, The Netherlands; 2 Faculty of Veterinary Medicine, Utrecht University, Yalelaan 1, 3508 TD Utrecht, The Netherlands

## Abstract

Interleukin 17 (IL-17) stimulates the osteogenic differentiation of progenitor cells *in vitro* through a synergy with bone morphogenetic protein (BMP)-2. This study investigates whether the diverse responses mediated by IL-17 *in vivo* also lead to enhanced BMP-2-induced bone formation. Since IL-17 is known to induce osteoclastogenesis, we studied the interactions between IL-17 and BMP-2 in ceramic scaffolds either or not carrying a coating with the bisphosphonate zoledronic acid (ZOL). Histological evaluation revealed that IL-17 alone did not induce any osteoclasts at day 10. On the other hand, BMP-2 clearly stimulated early tissue ingrowth and osteoclastogenesis. Both of these processes were blocked in presence of ZOL. IL-17 signaling restored early vascularized connective tissue formation and osteoclastogenesis induced by BMP-2 in ZOL-coated scaffolds. After 12 weeks, the bone volume induced by co-delivery of BMP-2 and IL-17 was doubled as compared to that induced by BMP-2 alone. We conclude that IL-17 has osteo-stimulatory effects through a synergy with bone-inductive BMP-2. Although local and single application of IL-17 does not mediate osteoclast formation, it could promote other processes involved in bone formation such as connective tissue ingrowth. The use of IL-17 may contribute to the development of improved bone graft substitutes.

## Introduction

There is a need for bone substitutes that can decrease the reliance on autologous bone grafts for repair of bone defects^1^. An effective bone substitute consists of an osteoconductive matrix incorporated with biological factors that maximize the osteogenic response^2^. Bone morphogenetic protein 2 (BMP-2) is regarded as the factor with the highest potential for bone induction, but its current use is considered to be inefficient and unpredictable^3^. The clinical BMP-2 dose (1-2 mg/cc carrier)^4^ far exceeds the native concentration of BMPs, i.e. in the order of ng/cc bone^5^. Serious side effects have been reported for BMP-2, such as ectopic bone formation, osteolysis, and even urogenital or neurological complications^6^. These side effects are likely related to its supraphysiological dose^7^, however attempts to lower the clinical dose also resulted in significantly reduced bone augmentation^4,8^. There is evidence that sequential or simultaneous delivery of BMP-2 with other pro-osteogenic stimuli may increase the responsiveness of cells to BMP-2. For instance, a biological effect of BMP-2 can already be seen for a concentration as low as 25 ng/ml following co-delivery with multipotent mesenchymal stromal cells (MSCs)^9^. Likewise, pro-inflammatory cytokines are thought to modulate bone regeneration in part through their interaction with BMP-2, suggesting that their co-delivery could potentiate the osteoinduction provided by BMP-2^10,11^.

This study investigates a potential role of the pro-inflammatory cytokine interleukin 17 (IL-17) as a modulator of BMP-2-induced new bone formation. IL-17 is mainly produced by T lymphocytes and neutrophils, and is an important mediator of the inflammatory response in conditions such as trauma, infection and autoimmunity^12^. In bone research, IL-17 has been implicated in the progression of inflammation-mediated bone loss^13,14^. On the other hand, IL-17 may also show pro-osteogenic effects. For example, IL-17 promotes bone healing and is a mediator of pathological bone formation in conditions such as spondyloarthritis^15–18^.

The mechanisms responsible for the pro-osteogenic effects of IL-17 are largely unknown. To our knowledge, only a single study has investigated the effect of exogenous IL-17 delivery on bone regeneration. This study showed attenuated calvarial defect healing when IL-17 was added to ceramic bone grafts^19^. Although this suggests that IL-17 exerts a negative effect on the osteogenic response, its influence on bone regeneration could be heavily dependent on the context of IL-17 signaling. First, IL-17 alone is not osteoinductive in MSC cultures, yet it dramatically increases MSC matrix mineralization mediated by BMP-2^18^. The investigation of possible synergistic actions between IL-17 and BMP-2 on *in vivo* osteogenesis is therefore of interest. Second, IL-17 signaling could be a double-edged sword in bone formation due to its stimulatory effects on osteoclast differentiation^13,20,21^. While a balanced osteoclast activity is normally coupled to osteoblast formation^22,23^, exaggerated osteoclastogenesis results in bone resorption and has a negative impact on new bone formation^14,24^. Therefore it is of relevance to determine if suppression of osteoclasts could skew IL-17-mediated effects towards enhanced bone formation. In the context of immunomodulatory strategies, the use of bisphosphonates such as zoledronic acid (ZOL) may be feasible, since they are widely used in clinical practice and have shown to enhance bone mass also when applied under inflammatory conditions^24,25^. The effect of IL-17 on osteoblastogenesis is studied in an ectopic location, where the formation of bone tissue relies completely on the recruitment and differentiation of MSCs towards the osteogenic lineage^26^. It has been shown that MSCs recruited from either bone marrow, circulation, or local vascularized tissue will home to bone scaffolds following ectopic implantation^27,28^. This methodology allows for better study of osteoinductive pathways compared to bone defect models, where native bone-inductive growth factors and cells support healing partially through an osteoconductive mechanism^26^. In addition to MSCs, macrophages are considered an important cell type contributing to new bone formation through local immune modulation, growth factor production, and/or providing osteoclastogenesis^23,29,30^.

In the present study, we investigate how IL-17 affects ectopic bone formation in ceramic scaffolds in rabbits. Following the observation that IL-17 acts in a synergistic manner with osteoinductive factors *in vitro*, it was hypothesized that IL-17 could potentiate new bone formation induced by BMP-2. Effects of IL-17 were studied with respect to early tissue response and subsequent bone formation. To assess the role of osteoclasts in IL-17-mediated responses, scaffolds were used that were either or not coated with ZOL.

## Material and Methods

### Preparation of *in vivo* implants

Porous biphasic calcium phosphate (BCP) blocks were used with a total porosity of 75 ± 5%. The material consisted of 20 ± 5% β-tricalcium phosphate (TCP) and 80 ± 5% hydroxyapatite by weight^31^. The mechanical and biological properties of this ceramic (i.e. BCP 1150) have been described in detail elsewhere^32,33^. The blocks were autoclaved at 121 **°**C and dried at 60 **°**C before *in vivo* use. The relatively large scaffold size of 10 × 10 × 10 mm is still suited for subcutaneous implantations, while allowing stimulatory and inhibitory effects within a range of early bone formation processes. Considering the diffusion limit for oxygen of 100–200 μm^34^, the tissue ingrowth was expected to slowly occur from the edge of the implants towards the center.

The concentrations of ZOL, BMP-2 and IL-17 in the BCP scaffolds were based on previous reports. The ZOL concentration was selected according to a study by Ichikawa *et al*.^35^, who demonstrated increased bone formation in ectopic β-TCP scaffolds due to ZOL. The current IL-17 concentration range (1.3–133 ng/ml) was based on our own *in vitro* study^18^, in which we showed that IL-17 most strongly stimulated osteoblastogenesis at 50 ng/ml within a tested 5–500 ng/ml concentration range. We previously identified a BMP-2 concentration which resulted in a limited bone area% of 5% after 12 weeks in the same rabbit model^10^. A comparable suboptimal BMP-2 dose was used in the current study to discriminate the positive or negative effects of the mediators of interest.

To prepare ZOL (Zometa®, Novartis Pharma, Switzerland)-coated scaffolds, 110 μl of a 228 μg/ml stock solution in MilliQ was pipetted onto the BCP blocks. This volume was completely absorbed by the scaffold. This resulted in an end concentration of 33.4 μg/ml, considering that 0.75 cm^3^ is the available space in the scaffolds. Control constructs received 110 μl MilliQ. The samples were air dried for 48 h at room temperature. Non-precoated and ZOL-precoated scaffolds were loaded with rhIL-17 (R&D Systems, MN, USA), alone or in combination with rhBMP-2 (InductOS®, Wyeth/Pfizer, NY, USA), according to Table 1. For the constructs containing BMP-2, first 30 μl of a 100 μg/ml BMP-2 stock solution in PBS was pipetted onto the scaffolds, resulting in an end concentration of 4 μg/ml in the scaffolds. The other constructs received 30 μl PBS. Subsequently, 80 μl of an IL-17 stock solution (12.5–1250 ng/ml) in PBS was pipetted onto the scaffolds to an end concentration of 1.3–133 ng/ml. The controls received 80 μl PBS. Implants were made on the day of surgery and stored in a humidified environment at 37 °C.

**Table 1:** Composition of the constructs for the *in vivo* assessment of 10-day tissue response and 12-week bone formation.

Group		BMP-2 (Dose)	IL-17 (Dose)	ZOL (Dose)	n
**10-day study**					
Non-coated scaffolds	Empty	—	—	—	3
	IL-17 high	—	100 ng	—	3
	BMP-2	3 μg	—	—	3
	BMP-2 + IL-17 high	3 μg	100 ng	—	3
ZOL-coated scaffolds	Empty	—	—	25 μg	3
	IL-17 high	—	100 ng	25 μg	3
	BMP-2	3 μg	—	25 μg	3
	BMP-2 + IL-17 high	3 μg	100 ng	25 μg	3
**12-week study**					
Non-coated scaffolds	Empty	—	—	—	4
	IL-17 high	—	100 ng	—	8
	BMP-2	3 μg	—	—	8
	BMP-2 + IL-17 low	3 μg	1 ng	—	8
	BMP-2 + IL-17 med	3 μg	10 ng	—	8
	BMP-2 + IL-17 high	3 μg	100 ng	—	8
ZOL- coated scaffolds	BMP-2	3 μg	—	25 μg	8
	BMP-2 + IL-17 low	3 μg	1 ng	25 μg	8
	BMP-2 + IL-17 med	3 μg	10 ng	25 μg	8
	BMP-2 + IL-17 high	3 μg	100 ng	25 μg	8

BMP-2, bone morphogenetic protein 2; IL-17, interleukin 17; ZOL, zoledronic acid.

### ZOL *in vitro* release experiment

Non-coated and ZOL-coated scaffolds were incubated in 700 μl MilliQ in eppendorf tubes at 37 °C. At different time points, the scaffolds were removed and the supernatant was stored at −20 °C. Separate tubes were made for each time point in duplicate. The ZOL concentration in the supernatant was measured by High Performance Liquid Chromatography (HPLC) as described previously^36^. A mobile phase mixture was used consisting of methanol:water (10:90), with 6 mM tetrabutylammonium hydrogen as an ion pair for ZOL at pH 2.6. Samples were injected into the HPLC system (Alliance 2695, Waters Corporation, MA, USA) coupled with LiChrospher^®^ C-18 column (Merck Millipore, MA, USA). The absorption was measured at 208 nm (UV detector 2478, Waters) at an elution speed of 0.8 ml/min. Results were analyzed with Waters Empower 3 Pro software.

### Animal study

Animal experiments were performed after approval of the local Ethics Committee for Animal Experimentation (Utrecht University, Utrecht, The Netherlands). All procedures were carried out in accordance with the approved guidelines, and all efforts were made to minimize the number of animals used and their suffering. A total of twelve male New Zealand White rabbits (14 weeks old, 2.5-3.0 kg, Crl:KBL, Charles River, France) were used, and were housed at the Central Laboratory Animal Research Facility, Utrecht University. One rabbit from the 10-day study died the day after surgery and the cause of death could not be identified by necropsy. The implants from this rabbit were retrieved within an hour after its death for histological analyses (24 h constructs). The missing rabbit was replaced to match the group sizes. All other animals recovered well from the surgery and implanted samples were all retrieved. Three rabbits were euthanized after 10 days to study the early tissue response. Eight rabbits were euthanized after 12 weeks to study bone formation (Table 1).

The rabbits received ketamine (15 mg/kg *i.m*.; Narketan®, Vétoquinol, The Netherlands) and glycopyrrolate (0.1 mg/kg *i.m*.; Robinul®, Riemser Arzneimittel, Germany) pre-operatively, and medetomidine (0.25 mg/kg *s.c*.; Dexdomitor®, Orion Corporation, Finland) peri-operatively. Anesthesia was reversed with atipamezole hydrochloride (0.5-1.0 mg/kg *i.v*., Atipam®, Eurovet Animal Health, The Netherlands). Antibiotic prophylaxis (Penicillin 3 × 10^4^, IE benzylpenicilline/kg, Duplocilline®, Merck Animal Health, WI, USA) was given once peri-operatively. Animals received pain relief pre-operatively, and post-operatively every 12 hours for 2 days with buprenorphine (0.03 mg/kg *s.c.;* Temgesic®, RB Pharmaceuticals Limited, UK).

**Figure 1:**
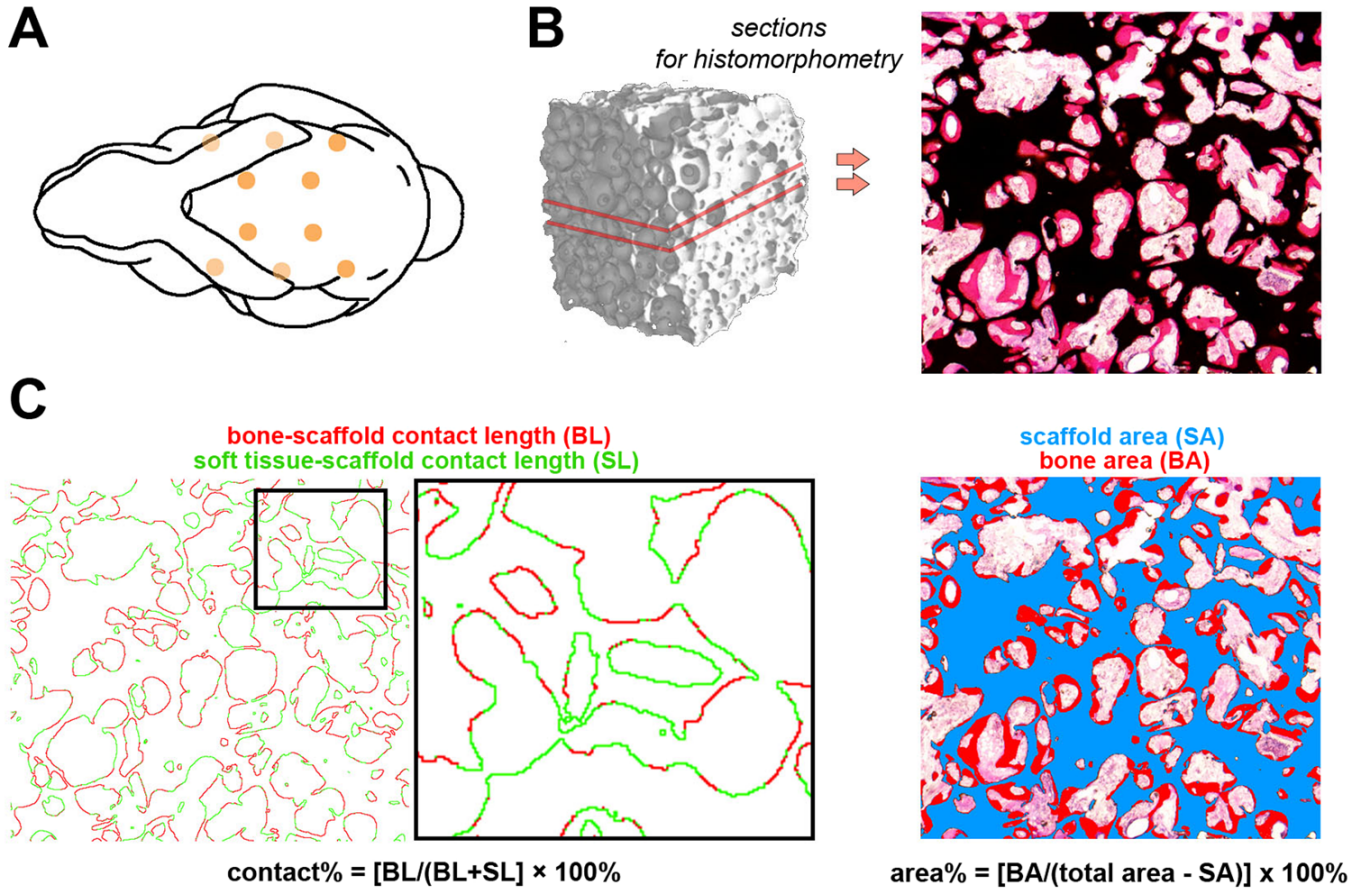
Quantification of bone by histomorphometry. (**A**) Schematic representation showing the predefined subcutaneous implantation locations in the rabbit dorsum. (**B**) The bone tissue was stained with methylene blue/basic fuchsin on two MMA midsections. (**C**) The bone and scaffold material were pseudo-colored for calculation of the bone contact% and bone area%.

After shaving and disinfecting the skin, subcutaneous pockets were created in the dorsum by incision of the skin and blunt dissection of the subcutaneous tissue. The animals received each of the conditions listed in Table 1, thus eight to ten constructs were implanted per animal. To circumvent that adjacent samples could influence the outcome in a systematic manner, the different scaffolds were allocated to one of the predefined subcutaneous implantation locations (Fig. 1A) with an online randomizer tool. To ensure that the subcutaneous pockets were well separated with at least 4 cm spacing, half of the samples were implanted immediately adjacent to the spine, and the other half were implanted more laterally. The pockets were closed with a suture (Prolene®, Ethicon, NJ, USA). The rabbits were euthanized after 10 days (n = 3) or 12 weeks (n = 8) with a sodium pentobarbital injection (Euthanimal®, Alfasan, The Netherlands) after inducing the same general anesthesia as the surgery.

### Histological processing

Constructs were fixed in 4% (w/v) formaldehyde. For the 12-week study, a third of each sample was removed and decalcified in 0.3 M EDTA for paraffin embedding. The remaining material was embedded in methyl methacrylate. The constructs from the 10-day study were entirely decalcified and embedded in paraffin.

### Histological staining

Hematoxylin and eosin (H&E) staining was performed to evaluate general tissue morphology. To demonstrate connective tissue formation by Goldner’s trichrome staining, sections were deparaffinized and incubated with Weigert’s hematoxylin for 5 min, followed by Goldner’s solution [0.2% (v/v) glacial acetic acid, 0.033% (w/v) acid fuchsin, 0.13% (w/v) Ponceaux de xilidine] for 45 s, 2% (w/v) Orange G for 7 min and 0.15% (w/v) Light green for 7 min. Differentiation was done with 1% (v/v) acetic acid for 5 s after each staining. Samples were dehydrated and mounted in Depex. Sections were scored by two blinded observers for presence of connective tissue in either 0, 25, 50, 75 or 100% of the available space. Data are presented as the average of the two scores.

For detection of osteoclasts, staining for tartrate-resistant acid phosphatase (TRAP) activity was performed. Samples were incubated with 0.2 M acetate buffer-tartaric acid for 20 min at 37 °C. Naphtol AS-MX phosphate (0.5 mg/ml, Sigma, MO, USA) and Fast red TR salt (1.1 mg/ml, Sigma) were added for another 2 h. Sections were counterstained with Mayer’s hematoxylin. Osteoclasts were defined as multinucleated TRAP-positive cells lining the surface of bone or scaffold. An absolute osteoclast count was considered to be a reliable measure of osteoclastogenesis since it correlates with *in vitro* resorptive activity^37^. The number of osteoclasts was counted in one cross-section from each construct and averaged per group.

### Immunohistochemistry

Stainings for calprotectin-expressing phagocytes (clone MAC387, Bio-Rad, CA, USA) or T lymphocytes (CD3, clone F7.2.38, Dako, Denmark) were performed as described previously^10^. The number of positive cells was counted in one cross-section from each construct and averaged per group.

To stain blood vessels, samples were incubated with anti-CD31 (clone JC70A, 1:50, Dako) for 1 h at room temperature following heat-induced antigen retrieval with EDTA (1 mM, pH 9.0). Blocking and staining was performed with a kit, according to the manufacturer’s protocol (Dako EnVision + System HRP, Dako).

Using the same kit as the CD31 staining, cytoplasmic staining for connective tissue macrophages (clone RAM11, 1:100, Dako) was performed with overnight incubation of the primary antibody at 4 °C. Sections were counterstained with Mayer’s hematoxylin.

### Bone histomorphometry

X-ray based imaging techniques, including high-resolution micro-CT, were considered unreliable methods to quantify the bone volume due to the artifacts caused by the BCP material^38,39^. Bone histomorphometry on MMA sections was therefore the best representative of the bone volume present in the scaffolds. Two MMA midsections were cut with a saw microtome (Leica, Germany) at 35 μm thicknesses and stained with basic fuchsin/methylene blue (Fig. 1B). The bone and scaffold were pseudo-colored in Adobe Photoshop (Adobe Systems, CA, USA). This allowed quantification of the percentage of bone in the available pore space (bone area %), or percentage of the scaffold surface in contact with bone (bone contact%) (Fig. 1C). The mean value of two sections per scaffold was used for further analyses.

### Statistical analyses

A sample size calculation was performed for the 12-week study, using the bone area% as main outcome parameter. A power of 80% was used, with an alpha of 5% that was adjusted for multiple comparisons. This indicated that a sample size of 8 was needed to detect a 50% bone volume change given a 30% standard deviation. Changes in bone volume were analyzed using a linear mixed-model approach in SPSS (IBM, IL, USA). The animals received each of the conditions listed in Table 1. To take into account animal-specific effects, pairwise comparisons were made between each IL-17 concentration and the control implanted in the same animal. Bonferroni correction was used for post hoc analysis. An arbitrary sample size of 3 was chosen for the 10-day study. All results are shown as the mean ± standard deviation (SD).

### Data availability

The datasets generated during the current study are available from the corresponding author on reasonable request.

## Results

### ZOL release from ZOL-coated scaffolds

**Figure 2:**
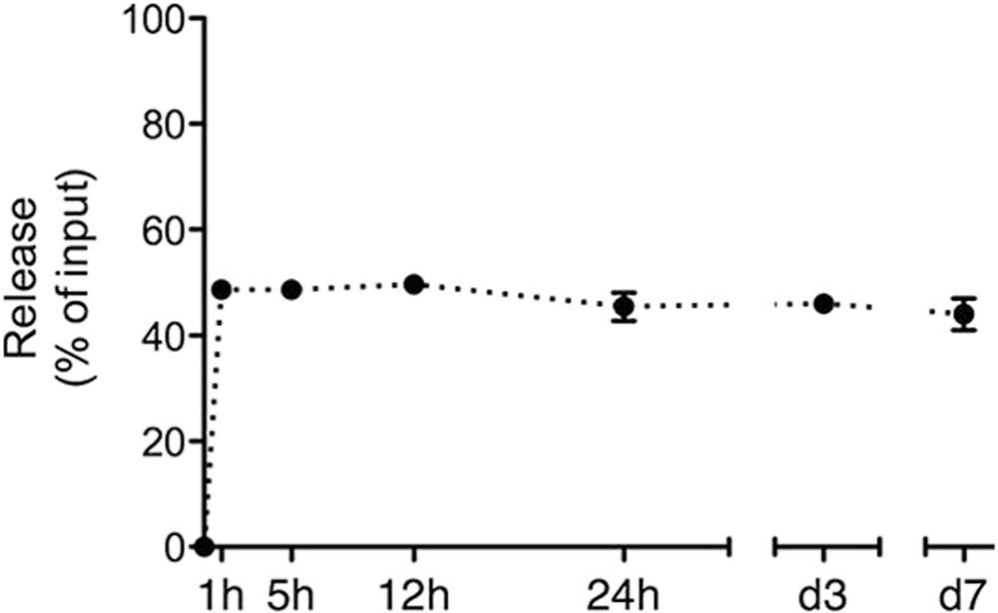
*In vitro* release of zoledronic acid (ZOL) from BCP scaffolds. Scaffolds precoated with 25 μg ZOL were incubated in MilliQ at 37 °C. The released amount of ZOL was measured in the supernatant at the indicated time points and normalized to the input. The values represent the mean ± SD for duplicate measurements.

*In vitro* release measurements showed that the scaffolds retained approximately half of the loaded ZOL after an initial burst release, without any additional release at later time points (Fig. 2). Hence, the ZOL was strongly bound to the BCP, which corresponds with its high affinity for hydroxyapatite^40^.

### Early tissue response

**Figure 3:**
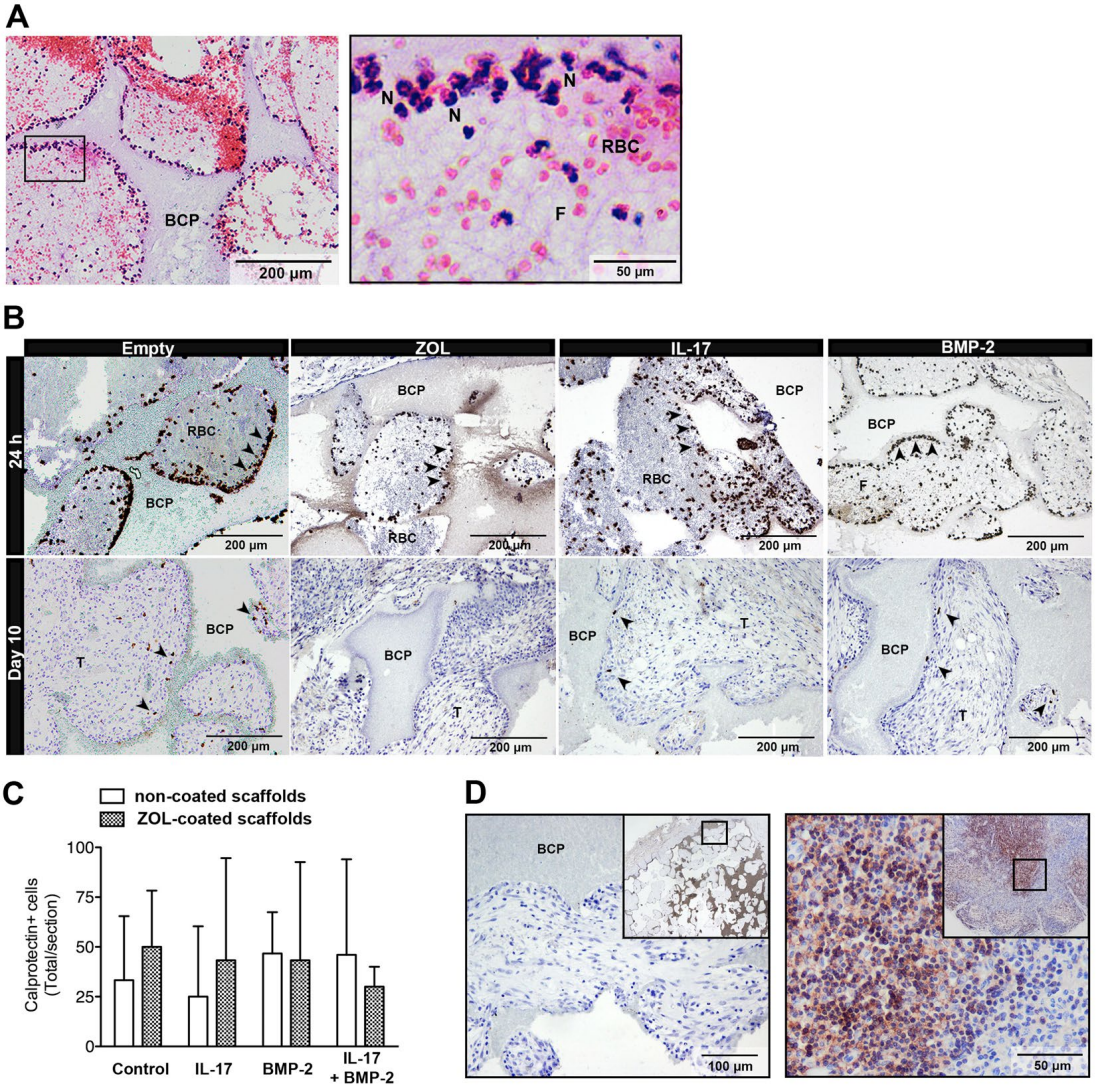
Inflammatory response after 24 hours and 10 days. (**A**) H&E staining was performed on an empty scaffold retrieved after 24 days. This sample is representative for all 24 h samples. (**B**) Calprotectin staining (in brown, arrowheads) was performed to demonstrate activated phagocytes in the constructs after 24 h or 10 days. (**C**) Calprotectin-positive cell count at day 10, represented as the mean ± SD (n = 3). (**D**) CD3 staining for T lymphocytes was performed on an empty scaffold retrieved after 10 days (left panel). This sample is representative for all day 10 samples. A rabbit lymph node was used as a positive control (right panel). The top right insets provide an overview. BCP, biphasic calcium phosphate; F, fibrin network; N, neutrophils; RBC, red blood cells; T, connective tissue.

The 24 h constructs contained mainly erythrocytes and calprotectin-expressing neutrophils within a fibrin network (Fig. 3A,B). In comparison, calprotectin-expressing phagocytes were relatively scarce in the 10-day constructs, and appeared to be macrophages based on their morphology (Fig. 3B). The studied mediators did not seem to promote active inflammation at day 10, considering the similar number of calprotectin-expressing cells in the different groups (Fig. 3C). Staining for CD3 showed an absence of T lymphocytes (Fig. 3D).

**Figure 4:**
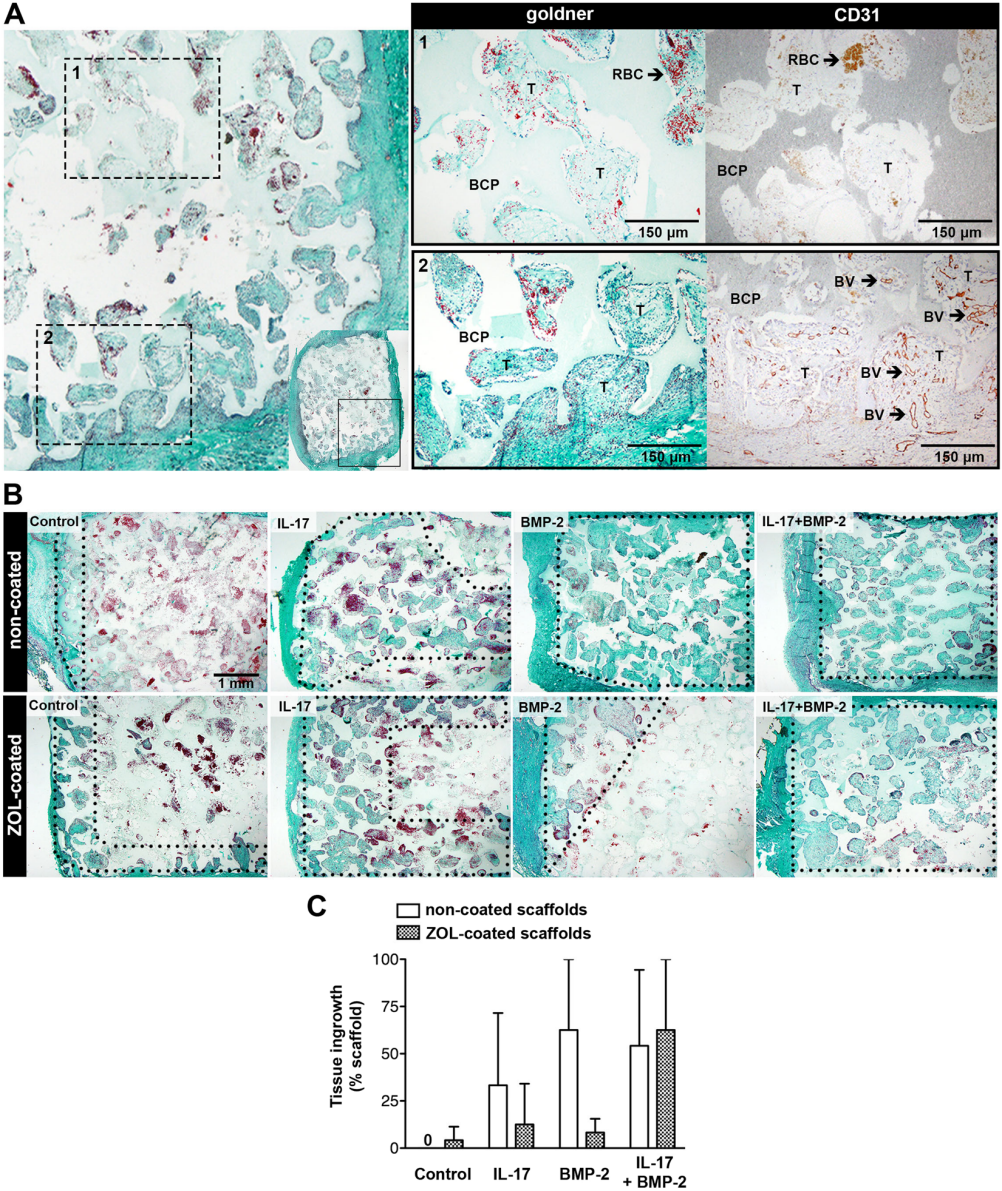
Connective tissue ingrowth in the 10-day study. (**A**) Goldner’s trichrome staining was indicative of collagen in connective tissue (in green); note differences in collagen between the periphery and central regions in a BMP-2-loaded construct without ZOL. CD31-positive blood vessels (BV, in brown) were only found in regions where connective tissue (T) had formed. (**B**) Goldner’s trichrome staining showing differences in connective tissue formation between groups. A region of interest is shown which covers half of the construct’s total area from the periphery (left) to the center (right). The areas within the dotted lines indicate regions of vascularized connective tissue formation. All images are representative for the group. (**C**) Quantification of vascularized connective tissue ingrowth, represented as the mean ± SD (n = 3). BCP, biphasic calcium phosphate; T, connective tissue; RBC, red blood cells.

**Figure 5:**
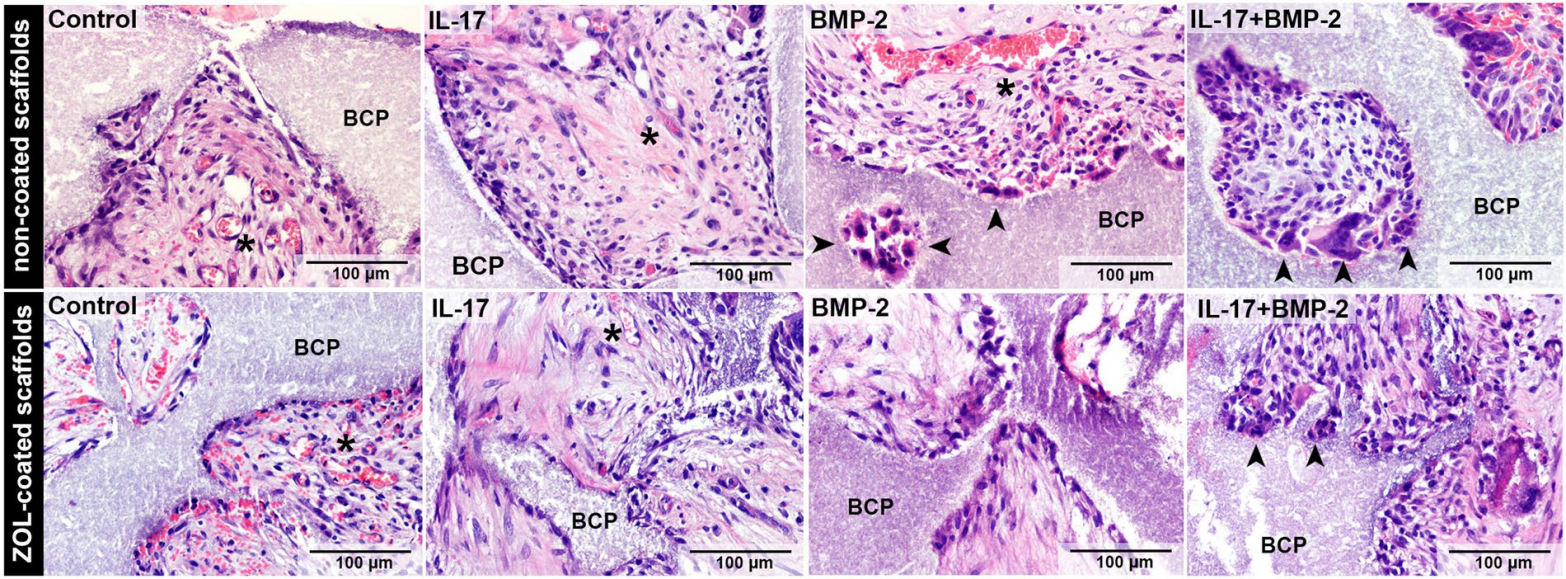
Day 10 H&E staining. Images are taken from the edge of the scaffold and are representative for the group. Arrowheads point to multinucleated cells indicative of osteoclasts. Asterisks indicate blood vessels. BCP, biphasic calcium phosphate.

The combination of Goldner’s trichrome and CD31 stainings showed that the ingrowth of vascularized connective tissue was not completed in all constructs by day 10, and instead, remnants of the hematoma and necrotic cells were prominent in the central regions of many constructs (Fig. 4A). Vascularized connective tissue formation was enhanced by BMP-2 and IL-17, and was abrogated in ZOL-coated constructs (Fig. 4B,C). This points towards an inhibitory effect of ZOL on early connective tissue ingrowth. Co-administration of IL-17 and BMP-2 was not sensitive to the inhibitory effect of ZOL. Goldner’s trichrome staining indicated no formation of osteoid or bone in any of the constructs at this time point. H&E confirmed the absence of osteoid (Fig. 5). Furthermore, in BMP-2-loaded groups, numerous multinucleated cells resembling osteoclasts were seen lining the BCP surface (Fig. 5).

**Figure 6:**
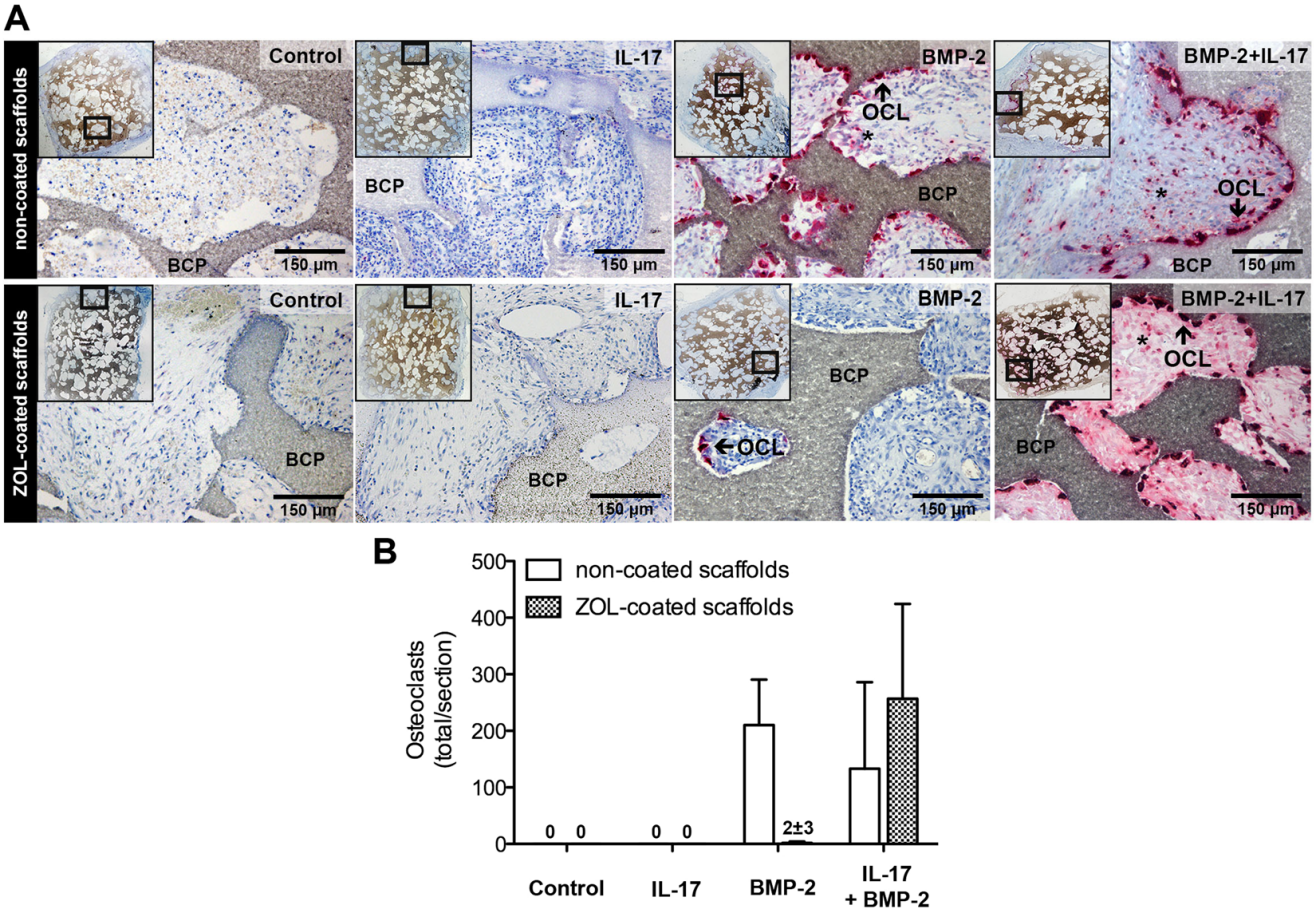
Role of osteoclasts during the early tissue response. (**A**) Tartrate-resistant acid phosphatase (TRAP) staining shows day-10 presence of osteoclasts (OCL, in red) and mononucleated TRAP-positive cells (indicated by asterisks). ZOL-coated and non-coated biphasic calcium phosphate (BCP) scaffolds were left untreated (control), or were loaded with IL-17 (100 ng/construct), BMP-2 (3 μg/construct), or their combination. Representative images of each group are given, with the scaffold overview in insets. (**B**) Osteoclast counts are represented as the mean ± SD (n = 3). Mononucleated TRAP-positive cells were not included in the osteoclast counts.

The 10-day time point coincides with an initial peak in osteoclast formation in osteogenic constructs^23,41^. Analysis of the number of osteoclasts showed a complete absence in the empty controls. Osteoclasts were neither detected in samples with IL-17 alone. In contrast, many osteoclasts were present in BMP-2-loaded scaffolds without ZOL, which were preferentially localized at the periphery of the scaffolds (Fig. 6A). The induction of osteoclasts by BMP-2 was efficiently prevented in ZOL-coated scaffolds (Fig. 6B). Similar to the established trend for vascularized connective tissue ingrowth, co-administration of IL-17 and BMP-2 was insensitive to the inhibitive effects of the ZOL-coating, and resulted in a large presence of osteoclasts.

**Figure 7:**
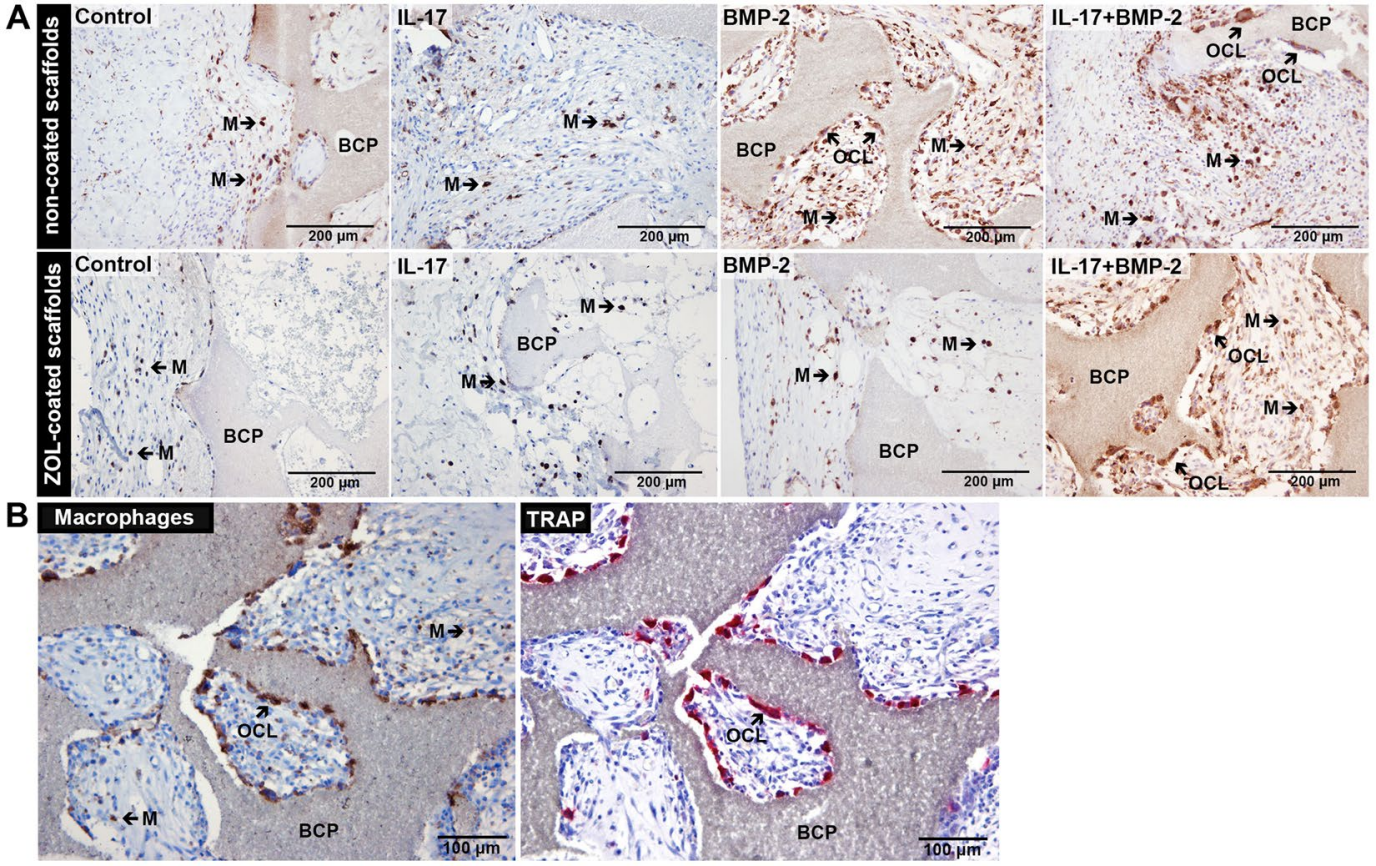
Presence of macrophages at day 10. Zoledronic acid (ZOL)-coated and non-coated biphasic calcium phosphate (BCP) scaffolds were loaded with BMP-2 (3 μg/construct) and/or IL-17 (100 ng/construct) and implanted subcutaneously for 10 days. (**A**) Images taken from the edge of the constructs stained with the RAM11 antibody (brown) show the presence of connective tissue macrophages (M) and multinucleated cells resembling osteoclasts (OCL). (**B**) Consecutive sections showing the co-localization of multinucleated RAM11-positive and tartrate-resistant acid phosphatase (TRAP)-positive osteoclasts (OCL). Mononucleated RAM11-positive cells indicate connective tissue macrophages (M). Images are representative for BMP-2-loaded constructs without ZOL.

A staining for connective tissue macrophages was performed to determine whether the ZOL coating also affected TRAP-negative phagocytes. Macrophages were found in the periphery of the constructs in all conditions, irrespective of ZOL-coating, but were more frequent in samples demonstrating enhanced vascularized connective tissue ingrowth (Fig. 7A). The highest density of macrophages was seen in regions demonstrating increased osteoclastogenesis (i.e. BMP-2 + IL-17 conditions), with co-localization of TRAP and macrophage stainings in serial sections (Fig. 7B). No quantitative analysis could be performed due to sample detachment in the center of the scaffolds as a result of the RAM11 staining protocol.

### The effect of IL-17 on new bone formation

**Figure 8:**
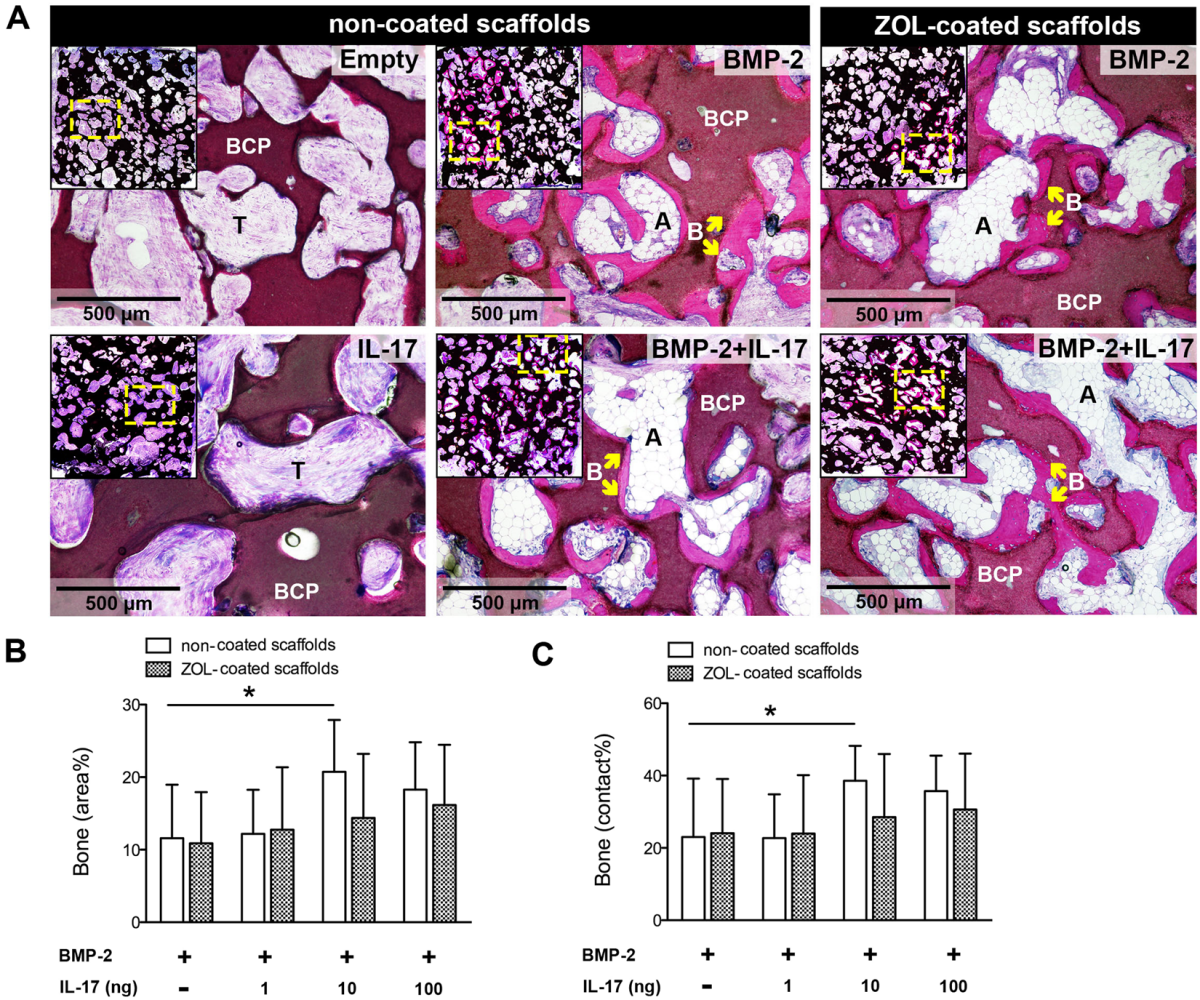
Bone formation in scaffolds with suboptimal BMP-2 dose after 12-week implantation (**A**) Methylene blue/basic fuchsin staining of bone (**B**, pink), with the scaffold overview in insets (**B**,**C**) Quantification of bone formation as bone area% (**B**) and bone contact% (**C**). Data are represented as the mean ± SD (n = 8). *p < 0.05. BCP, biphasic calcium phosphate; A, adipose tissue; T, connective tissue.

There were no signs of BCP degradation during the 12-week implantation. All constructs showed complete vascularized connective tissue ingrowth. Empty BCP constructs or constructs that contained IL-17 (100 ng/construct) did not contain any bone. The groups that contained BMP-2 demonstrated the presence of dense lamellar bone and adipose tissue (Fig. 8A). BMP-2 was chosen at the suboptimal dose of 3 μg/construct to enable detection of IL-17-mediated effects in both directions. A dose-dependent increase in the amount of bone was seen when IL-17 was co-administered (Fig. 8B). The optimal condition with 10 ng IL-17/construct significantly enhanced the bone area% from 11.6 ± 7.4 to 20.3 ± 7.1 (p = 0.042). For BCP constructs coated with ZOL, increased bone formation with IL-17 was not significant. Analysis of bone contact% showed the same trend as the area% (Fig. 8C): with only BMP-2 it was 23.0 ± 16.2, and increased to 35.7 ± 9.8 when 10 ng IL-17/construct was co-administered (p = 0.016).

### Immune response at week 12

**Figure 9:**
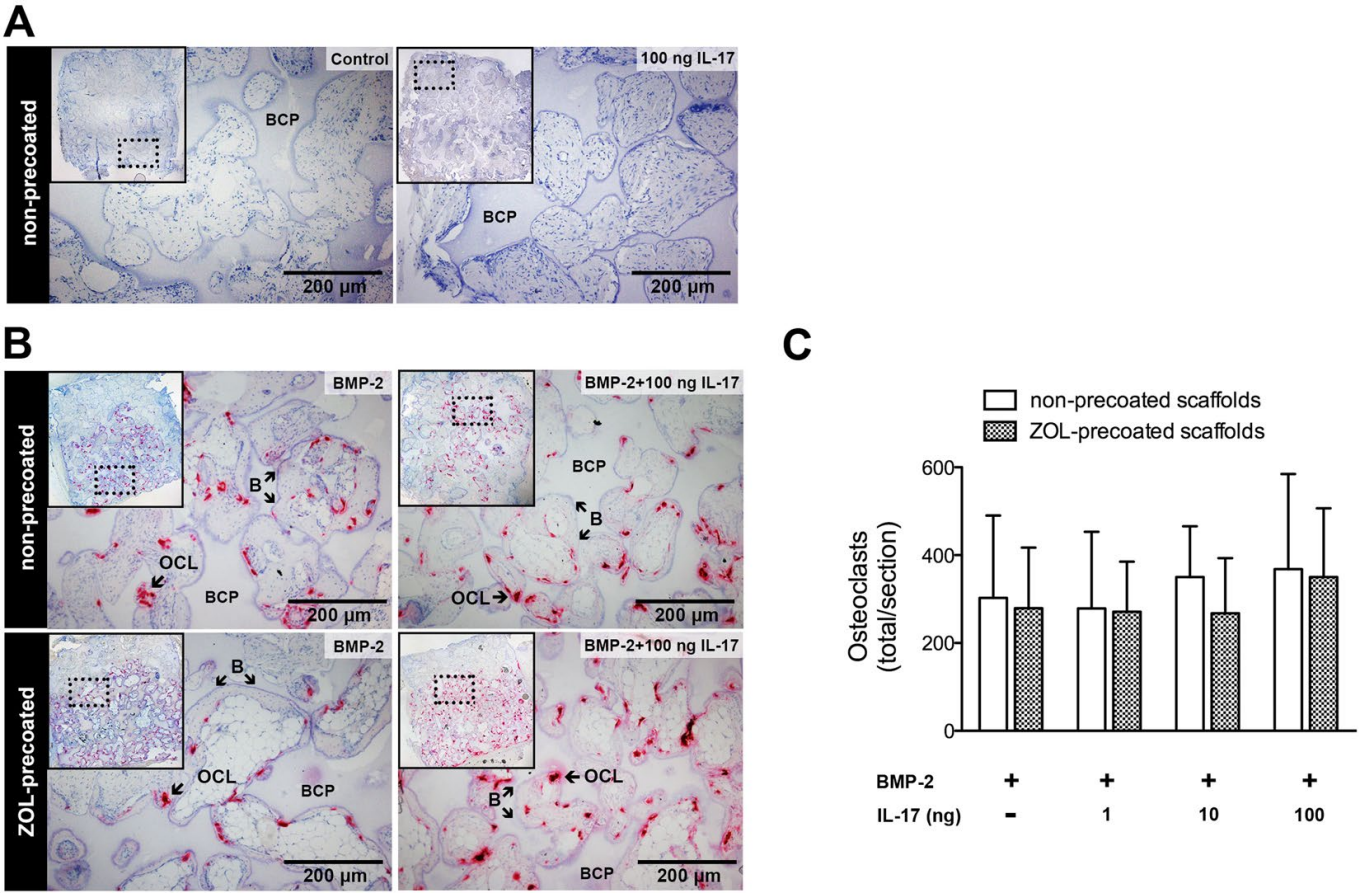
Presence of osteoclasts in the newly formed bone after 12-week implantation. (**A**) Tartrate-resistant acid phosphatase (TRAP) staining for osteoclasts was performed on empty biphasic calcium phosphate (BCP) scaffolds (control) or scaffolds loaded with IL-17 (100 ng/construct). (**B**) ZOL- coated and non-coated scaffolds were loaded with BMP-2 (3 μg/construct) with or without IL-17 (100 ng/construct) and implanted subcutaneously. TRAP staining shows the presence of bone (B)-lining osteoclasts (OCL, in red). The images for this IL-17 concentration are representative for all IL-17 concentrations. (**C**) Osteoclast counts are represented as the mean ± SD (n = 8).

No TRAP-positive cells were observed in empty scaffolds or scaffolds loaded with 100 ng IL-17 (Fig. 9A). Numerous TRAP-positive osteoclasts were found in BMP-2-loaded constructs, and were consistently localized at the bone surface (Fig. 9B). Their absolute numbers were comparable in the different groups (Fig. 9C). Calprotectin staining demonstrated an absence of activated phagocytes at this time point.

## Discussion

The objective of this study was to explore the possible role of IL-17 in the early and/or late osteogenic response. BCP scaffolds with a suboptimal amount of BMP-2, either or not combined with IL-17, were investigated in subcutaneous pockets in rabbits. In this location, IL-17-mediated effects can be detected on osteoinductive pathways leading to bone formation. In line with its promotive effect on MSC osteogenic differentiation^18^, IL-17 significantly enhanced BMP-2-induced new bone formation. We furthermore showed that IL-17 does not induce early osteoclastogenesis, but likely supports other processes involved in bone formation including vascularized connective tissue ingrowth.

In the current study, the combined delivery of BMP-2 and IL-17 led to a twofold greater bone volume as compared to BMP-2 alone. Our observation that IL-17 enhances bone formation contradicts the study by Kim *et al*., who found an inhibitory effect of IL-17 on calvarial bone defect healing in rats. They, and others, supported this *in vivo* finding by showing decreased osteogenic marker expression in calvarial osteoblasts *in vitro*^19,42^. As an explanation, the effects of IL-17 on bone formation could be dependent on the cells that are targeted in that specific model. We found a stimulatory effect of IL-17 on ectopic new bone formation, i.e. a process that relies on the migration and differentiation of inducible cells^28^. In agreement, IL-17 potently enhances further osteogenic differentiation of undifferentiated MSCs and other immature cells^15,18,43,44^. Thus, IL-17 may have different effects on bone cells depending on their differentiation stage. Furthermore, the IL-17 receptor expression levels may vary in bone cells isolated from various sources, which could explain variable efficacy of IL-17 depending on the target tissue^19,43^. In the current study, only IL-17A was investigated, commonly referred to as IL-17^45^. Other IL-17 family members may also be interesting to study, since we already confirmed a pro-osteogenic role for IL-17F *in vitro*^18^.

The observed synergistic interaction between IL-17 and suboptimal dose BMP-2 in terms of bone formation may recapitulate the natural crosstalk between pro-inflammatory and osteogenic signaling pathways during bone regeneration^11^. IL-17 was effective in the ng/ml range, resembling its expression levels during inflammation^46^. Furthermore, an *in vivo* burst release of IL-17 was expected, which mimics its short-lived action in the bone healing response^15^. The timely delivery of a physiological amount of IL-17 could be a potentially feasible method, as it circumvents the destructive effects of chronic IL-17 signaling^14,45^. Consequently, the co-delivery approach should be explored as a strategy to improve the efficacy of BMP-2. This is of great importance, since the high costs and side effects of BMP-2 are likely related to its inefficient use^3^.

To further establish the potential of IL-17, it is of relevance to determine if its pro-osteogenic effects can also augment the healing of bone defects. As such, ectopic bone models neglect parameters that will ultimately direct the bone healing response in clinical scenario’s, such as presence of bone-stimulating cytokines, bone-forming cells, or bone-mechanotransduction^47^. It is therefore crucial to evaluate whether IL-17 can enhance the bone mass and mechanical stability in functional models such as spinal fusion or critical-size defects^48–50^. It is furthermore of interest to study how IL-17 modulates the osteoconductive and/or osteoinductive properties of ceramics with a faster resorption rate, including certain TCP formulations, since they usually promote better healing in large bone defect models than the currently studied BCP^33^. As part of these studies, fluorochrome labeling could provide valuable insight in the location and dynamics of new bone deposition relative to the existing bone^51^.

In line with other observations^23,41^, we found that the presence of high numbers of osteoclasts precedes new bone formation in the osteogenic constructs. To test the hypothesis that IL-17-mediated effects on osteoclastogenesis hampers its pro-osteogenic effects^24^, IL-17 in combination with the osteoclast-inhibitor ZOL was studied. To this end, a local ZOL dose (25 μg/scaffold) was applied that was considered clinically-relevant, as the systemic dose used to treat bone loss is approximately 60 μg/kg^52^. Consequently, the ZOL coating was found to be very effective in blocking osteoclast presence in the BMP-2-stimulated condition. In contradiction to the hypothesis, there was no obvious stimulatory effect of IL-17, alone or combined with BMP-2, on the number of osteoclasts. Furthermore, changes in early osteoclast activity did not necessarily affect the osteogenic response. Whereas osteoclasts play a prominent role in cell- or material-induced ectopic bone formation^23,29^, we found similar BMP-2-mediated bone formation ZOL-coated scaffolds as compared to non-coated scaffolds. In addition, others have shown that appropriate timing and/or administration route of ZOL treatment can lead to more favorable bone formation by preventing osteolysis caused by supraphysiological dose BMP-2^35,53–55^. We conclude that the pro-osteogenic effects of IL-17 cannot be convincingly attributed to changes in osteoclastogenesis.

Alternatively, we provide evidence that other early processes critical for bone formation may be positively influenced by IL-17. As part of the TGF-β superfamily, BMP-2 plays a role in tissue repair by stimulating cell recruitment, MSC proliferation/differentiation and extracellular matrix production^11,56^. The vascularized connective tissue ingrowth seen at day 10 in constructs with BMP-2 was strongly impaired by ZOL. Several mechanisms could be responsible for this inhibitory effect, depending on the ZOL concentration and timing. For instance, ZOL inhibits blood vessel formation induced by vascular endothelial growth factor (VEGF) and has pro-apoptotic effects on endothelial cells. Equally important, ZOL has negative influences on the survival and function of bone (precursor) cells depending on the applied concentration^57,58^. The negative impact of ZOL on BMP-2-induced vascularization and connective tissue formation were counteracted by IL-17. In line with these findings, IL-17 contributes to wound healing through the recruitment of other inflammatory cells and the modulation of angiogenesis^59^. MSCs could be a particularly important target, showing changes in migration, proliferation/differentiation and cytokine expression in response to IL-17^43,60–62^. Hence, the identification of the different targets of IL-17 in the osteogenic response is an important focus of further investigation.

The induction of IL-17 is limited to the first few days after bone injury^15^. In line, we found a synergistic interaction between IL-17 and BMP-2 using a burst release approach. It remains of interest to establish whether timing is important with regard to the osteogenic response enhanced by IL-17. It is generally thought that prolonged pro-inflammatory cytokine signaling impairs bone formation through several mechanisms, including decreased extracellular matrix production, delayed angiogenesis and/or uncontrolled cell death^63,64^. When studying prolonged IL-17 signaling using a long-term release system^65^, several systemic parameters are increasingly important to take into account. First, possible detrimental effects of chronic IL-17 signaling on systemic inflammation or generalized bone loss should be closely evaluated^66^. Second, detection of circulating IL-17 levels could exclude possible interference with distant sites. As the studied mediators all have a relatively short systemic half-life (minutes-hours)^67–69^, it was estimated that the incorporated factors had no or very limited effects on other ectopic sites.

Several recommendations can be made for future work. Initially, relatively few experimental animals were used to explore the effects of IL-17 with respect to early tissue response. Accordingly, the reported direction and effect size induced by IL-17 and/or ZOL on the observed histological changes will allow for more precise sample size calculations in future quantitative studies. Furthermore, due to incomplete connective ingrowth in the day-10 samples and tissue loss during processing, the center of the scaffolds could not be evaluated for CD31 and RAM11 expression. For the same reason, reliable immunodetection of osteoblasts was not possible at early time points. Scaffold homogenization and measurement of bone markers at the mRNA level could overcome this issue in the future^29^. In addition, cell isolation and detection of specific cell populations by fluorescence activated cell sorting could for example provide valuable information regarding the osteoblast/osteoclast ratio’s in the scaffolds^15^.

In the present study, we identify a novel role of IL-17 in potentiating bone substitutes. It was observed that IL-17 acts in a synergistic manner with BMP-2 to enhance new bone formation, but the mechanism by which IL-17 mediates this remains elusive. In addition to its stimulatory effect on MSC osteogenic differentiation, we showed that IL-17 may promote soft tissue ingrowth. On the other hand, IL-17 does not promote osteoclastogenesis. Together, these findings suggest that the induction of IL-17 signaling is a feasible strategy to promote bone regeneration.
